# Shared residential placement for child welfare and juvenile justice youth: current treatment needs and risk of adult criminal conviction

**DOI:** 10.1186/s13034-020-00355-1

**Published:** 2021-01-21

**Authors:** Lena Jäggi, Marc Schmid, David Bürgin, Nadine Saladin, Alexander Grob, Cyril Boonmann

**Affiliations:** 1 Division of Personality and Developmental Psychology, Department of Psychology, University of Basel, Basel, Switzerland; 2 Child and Adolescent Psychiatric Research Department, Psychiatric University Hospitals, University of Basel, Wilhelm Klein-Strasse 27, 4002 Basel, Switzerland; 3 Department of Forensic Child and Adolescent Psychiatry, Psychiatric University Hospitals, University of Basel, Basel, Switzerland

**Keywords:** Child welfare youths, Juvenile justice, Adult convictions, Trauma informed care, MAZ

## Abstract

**Background:**

Although child welfare youth and juvenile offenders in residential care have different judicial placement reasons, there seems to be overlap in their demographic and psychosocial backgrounds. This could raise the question whether these adolescents should be placed in strictly separated institutions based on their judicial title (civil or criminal law) or together based on their needs. As systematic knowledge on the effects of shared placement of these groups is limited, the aim of the current paper is to examine the demographic, crime-related and psychosocial characteristics of child welfare and juvenile justice youths in shared residential care and subsequently examine its relationship with offending behavior in adulthood.

**Methods:**

The sample was drawn from the Swiss study for clarification and goal-attainment in youth welfare and juvenile justice institutions (MAZ.) and consisted 354 juveniles (252 child welfare, 102 juvenile justice; 223 boys, 131 girls) between 10 and 18 years. Mental health problems were assessed with the Massachusetts Youth Screening Instrument-Version 2 (MAYSI-2), official adult criminal conviction data up to 10 years later was obtained from the Swiss Federal Office of Statistics. Three sets of logistic regressions were conducted investigating any, violent and non-violent convictions.

**Results:**

Univariate results showed that that the child welfare sample included more females, more juveniles with the Swiss nationality, and was younger at the time of assessment and at first placement compared to the juvenile justice sample. Furthermore, child welfare youths showed less alcohol/drug use problems and offending behavior than their juvenile justice counterparts. Unadjusted models demonstrated that committing authority predicted adult criminal convictions, but that this distinction disappeared when it was controlled for demographic, crime-related and psychosocial factors. Gender and time at risk were found to be related to adult conviction in all three models. In addition, alcohol/drug use problems were risk factors for general, previous convictions for violent, and traumatic experiences for non-violent convictions in adulthood.

**Conclusions:**

Our results support the approach of placement in residential care institutions based on treatment needs instead of on judicial title. Special attention should be devoted to trauma informed care and substance use coping. However, more research is needed.

## Background

Juvenile offenders in residential care are a population marked by highly elevated rates of trauma, psychopathology and other psychosocial problems [1, 2], while child welfare youth often show delinquent behavior in addition to similarly elevated rates of psychosocial treatment needs [3, 4]. Furthermore, a substantial number of juveniles involved with the juvenile justice system are so called crossover youth, meaning they have also been involved with child welfare authorities [5]. There have thus been political discussions to prioritize treatment needs and rehabilitation of juvenile offenders over punishment and not place them in juvenile correction facilities, but in the most appropriate residential treatment setting (e.g., [6]). This would involve sharing resources and institutions with child welfare youth who have been placed in out-of-home care because of, for example, maltreatment or neglect.

The Swiss juvenile justice system has an explicit focus on rehabilitation, education and treatment of delinquent juveniles [7]. In general, juvenile delinquency is viewed as a symptom for developmental adjustment problems and juvenile delinquents are viewed as a population in need of protection and guidance more than, and above, punishment alone. As in the adult system, there is a two-pronged approach separating punishment (“*Strafen”*) and interventions based on treatment needs (“*Massnahmen”*). For example, following a delinquent act, the court can order a juvenile to restitution or another punishment and/or, given the personal and social circumstances that might have contributed to the delinquent act (e.g., ongoing lack of parental supervision, mental health issues, developmental problems), an open-ended foster family or residential placement to address these issues [7]. This means minors can be placed in child welfare and juvenile justice institutions because of delinquent behavior (juvenile justice measure), child protection reasons (civil law measure, e.g., maltreatment, neglect, or parental absence, psychopathology or drug abuse) or other reasons (e.g., special needs, special education) [7, 8].

In Switzerland, out-of-home placement of children and juveniles is usually a measure of last resort, after other interventions within the family of origin have failed or a placement is deemed necessary to protect the child’s wellbeing and development [7, 9]. Children and adolescents are usually placed based on their age, gender and treatment needs and thus, unlike in many other countries, child welfare youth and juvenile delinquents can reside in the same facilities [8]. Switzerland thus offers an opportunity to study potential effects of shared placement of child welfare and juvenile justice youth. However, despite this ongoing practice, very little is known about minors placed in care in Switzerland in general [9], and there is no systematic knowledge on any effects of shared placement of juvenile offenders with child welfare youth to date.

The aim of the current paper is to map the demographic, crime-related and psychosocial characteristics of child welfare and juvenile justice youths in residential care in Switzerland, and subsequently examine its relationship with offending behavior in adulthood. This knowledge will not only inform us which factors to emphasize on in the assessment and treatment of these youngsters, but could also help better match the adolescents’ needs with the institution’s treatment options.

### Adolescents in out-of-home care in Switzerland

To this day, there are no official statistics on the total number of minors in foster care families or other out-of-home placement facilities in Switzerland, but estimates range from 22,000 to 30,000 children and adolescents [10]. Child protection is regulated by local authorities. Foster sector case management is less regulated and influenced by local structures, availability of local treatment options, and the individual qualifications of the case workers [9]. However, since the beginning of this century, steps have been taken to improve reporting, professionalism and quality control. In 2007, for example, a new juvenile criminal code (*Jugendstrafgesetz,* JStG) has entered into effect. All adolescents placed in a child welfare or juvenile justice institution through juvenile justice authorities have to be placed in an institution approved by the Swiss Federal Office of Justice (*Bundesamt für Justiz* [BJ]). To be approved, the institution have to fulfill certain quality and reporting standards [11] which, under new juvenile criminal law, includes regular assessments to document ongoing appropriateness of the placement [7]. In 2013, around 200 newly regionally consolidated professionalized and interdisciplinary Authorities for Child and Adult Protection (*Kindes- und Erwachsenschutzbehörden* [KESB]) have replaced the 1420 lay authorities which were organized on a municipal level [12]. At the same time, new federal legal regulations for foster placements (*Pflegekindverordnung* [PAVO]) have taken effect and the BJ has begun to collect and share best practices, general information and statistical analyses on youth placed in institutions or foster families on a new online platform (www.casadata.ch). The Swiss Federal Office of Statistics (*Bundesamt für Statistik* [BFS]) yearly publishes the number, age and gender of sentenced minors (e.g., [13]. Similarly, the National Conference for Child and Adult Protection (*Konferenz für Kindes- und Erwachsenenschutz* [KOKES]) now publish yearly numbers of child protection articles in court rulings (e.g., termination of parental rights or removal of custody), giving an idea of the number of new residential placements based on civil law. However, to date no information on the exact nature of the civil law intervention or any information on the children it aims to protect has been released.

### Mental health problems of youth in the child welfare system

A recent meta-analysis of studies from the U.S. and Europe showed that among children and adolescents in the child welfare system, 49% met criteria for a current mental disorder [3]. More in detail, 27% were diagnosed with a conduct (CD) or oppositional defiant disorder (ODD) and 11% met the criteria for an attention-deficit/hyperactivity disorder (ADHD). The population also had high rates of internalizing problems, with prevalence estimates for anxiety at 18%, depressive disorders at 11% and posttraumatic stress disorder (PTSD) at 4%, with higher prevalence of internalizing disorders among girls and more externalizing disorders among boys. In comparison, worldwide-pooled prevalence of mental health disorders within adolescents in the general population is estimated at 13%, with anxiety disorder at 7%, any disruptive disorder (i.e., CD or ODD) at 6%, ADHD at 3%, and any depressive disorder at 3% across geographic location [14].

These numbers have been even higher among youth placed in residential care, ranging from 49 to 76% [15–17], with high rates of comorbidity in all studies. Additionally, these adolescents show elevated rates of chronic illness [18], and childhood trauma [19], which in turn have been associated with worse mental health status into adulthood [20, 21]. For example, in a study with a Norwegian sample of youth in residential placement, the 71% of adolescents who had experienced maltreatment were even more likely to show CD, general anxiety disorder, dysthymia and major depressive disorder as well as more attempted suicides [22]. In addition, substance use as well as depression have been associated with increased rates of juvenile delinquency in youth in the child welfare system [4] and there is a substantial body of research linking past maltreatment experiences with delinquent behavior in adolescence [23, 24]; within the child welfare system, delinquency rates for youth with a history of maltreatment are approximately 47% greater than their non-maltreated counterparts [25]. In one recent U.S. study, for example, a history of maltreatment increased the risk of arrest by 55% and of committing a violent crime by 96% [26]. Finally, it is estimated that more than a third of youth in child welfare are known to the juvenile justice system [5, 24].

The limited data on Swiss samples of youth in out-of-home placements have not differentiated between child welfare and juvenile justice youth but have shown similar high rates of mental health treatment needs and high comorbidity, with overall 74% of children and adolescents fulfilling criteria for one, and 60% fulfilling criteria for more than one mental disorder in residential care populations [27]. Furthermore, 25% of them suffered from complex psychiatric disorders with emotional and behavioral symptoms and elevated rates of delinquency.

### Mental health problems of youth in the juvenile justice system

Similar to their counterparts in the child welfare system, youth in the juvenile justice system often come from backgrounds of poverty, family dysfunction, and maltreatment [28, 29]. Between 70 and 95% of detained youth were found to have mental health problems [1, 30, 31]. A meta-analysis of youth in juvenile detention and correctional facilities has shown that they are about 10 times more likely to suffer from psychosis than the general adolescent population [1], and there is a high prevalence of previous trauma and PTSD [32, 33]. Rates of substance use are extremely high, with dependence and abuse affecting between 40 and 70% of juvenile offenders in custody [31]. A systematic review among detained male adolescents found mean prevalence estimates of 70% for any mental disorders, with CD and substance use disorders (SUDs) being the most frequent [30]. Although their numbers are much smaller, girls in the juvenile justice system tend to be younger and have more severe mental health problems than their male counterparts [1, 34]. Fazel and colleagues [1] found that almost 30% of girls in detention qualified for a diagnosis of major depression compared to 11% of boys, almost 20% presented with ADHD, (12% among boys). Both girls and boys shared similar elevated rates of CD at 53% of the sample, but girls present with higher rates of comorbid externalizing and internalizing disorders. These findings are troubling, since meta-analytic results have shown that presenting with an externalizing disorder or comorbidity increases the risk of recidivism for juvenile delinquents by around 20% [35].

### Risk of adult criminal behavior

A substantial body of research links past maltreatment and neglect with juvenile delinquency and (violent) offending in adulthood [36]. Simultaneously, studies show an elevated risk of adult criminal involvement among former foster care youth [37, 38]. To date, the evidence regarding the influence of out-of-home placement on future delinquency is inconclusive [39, 40]. For example, it has been shown that any out-of-home placement increases the likelihood of delinquency in adolescence and into young adulthood, especially if there is a history of placement instability [25, 41, 42]. Type of placement matters as well, and for some adolescents, kinship or family foster care has been associated with better outcomes compared to residential placement in group homes [36]. However, all those findings are confounded by the fact that placement outside the home is usually reserved for the most severe cases of detected maltreatment, while more severe mental health, substance use and conduct problems are associated with both residential placement over family foster care and more placement instability, all of which independently influence likelihood of future delinquency [24, 38, 43–45]. Furthermore, a Swedish population-based study only found negative effects of placement in care on adult criminality for boys first placed between ages 13–18, but not for girls or younger boys [40].

Recent national data showed that 8% of adolescents born in Switzerland in 1992 were convicted as adolescents, and that having a juvenile conviction was associated with a six-fold increased odds of an adult conviction [13]. Among convicted Swiss adolescents, 26% were re-convicted as young adults. Additional analyses showed that being male, having multiple juvenile convictions, being over 16 years of age at first conviction as well as having been convicted for more severe crimes increased the risk of adult criminal conviction. However, the study did not differentiate between type of adult criminal conviction (violent or non-violent) and there is no knowledge on risk of adult criminal conviction among young adults who were in residential care as adolescents.

Based on the aforementioned information, the aim of the current study threefold. First, to examine similarities and differences in demographic markers, previous offending (self-report and official conviction) and treatment needs (psychiatric profile, substance use) between adolescents placed in residential care by either child protection or juvenile justice authority. Second, to investigate whether the adolescents committed by child protection or juvenile justice authority differ in their long-term risk for any, violent, and non-violent young adulthood criminal convictions. Third, to examine if this relationship persists after controlling for well-known risk factors for adult criminal conviction (gender, age at beginning of placement, trauma, past self-reported delinquency, past convictions) or mental health treatment needs in adolescent residential care.

## Methods

### Study procedures and sample description

The sample was drawn from the larger Swiss Study for Clarification and Goal-Attainment in Youth Welfare and Juvenile Justice Institutions, (*Modellversuch Abklärung und Zielerreichung in stationären Massnahmen* [MAZ.]) involving the standardized monitoring and evaluation of mental health problems of youths in child welfare and juvenile justice institutions throughout Switzerland between 2007 until 2012 [8], as well as criminal justice follow-up data until the end of 2017 In this study, all child welfare and juvenile justice institutions accredited by the BJ were invited to participate. The final sample of 64 participating facilities (35% of eligible facilities) were representative for the different types of institutions in Switzerland (e.g., large versus small institutions, institutions with or without internal schools, and internal versus external access to treatment programs) as well as the heterogeneous group of youths who reside in them. Adolescents who resided in one of the participating facilities were asked to participate if they had been living in the institution for more than one month prior to the assessment and were able to complete the French, German or Italian assessment tools. Assessments consisted of clinical interviews by trained psychologists, computer assisted self-reports as well as ratings from institutional caregivers. Hence, assessment was not conducted at entrance per se, but could also have been taken place after the adolescent was already in the institution for a while (*M* = 13.1 months; *SD* = 13.3).

Institutional staff approached adolescents and their legal guardians and explained the aims and nature of the study. A total of 592 (32%) adolescents from the participating institutions, in the French- (20 facilities), German- (38 facilities) and Italian-speaking (6 facilities) parts of Switzerland participated in the study. To check the representativeness of the study sample, institutional caregivers were asked to rate some adolescents who did not participate in the study (N = 46) on the Child Behavior Checklist (CBCL) [46] or the Young Adult Behavior Checklist (YABCL) [47]. Matched comparisons (i.e., on age and gender) between adolescents who did and did not participate in the study showed no differences in the frequency of scoring in the clinical range on the internalizing-, externalizing- and total problems scales of the CBCL or the YABCL. This suggests that the participating sample was representative for youth in the aforementioned participating institutions. For more details on study methodology see [8]. The Ethics Review Committees of Basel, Lausanne (Switzerland) and Ulm (Germany) approved the study.

### Participants

First, for the current study, adolescents had to be placed under either civil or criminal law. Second. participants under 10 years of age were excluded since under current Swiss law, there are no juvenile justice commitments before that age. Third, the upper limit was set at 18 years of age which is usually when child welfare placements end, and all participants placed in institutions for young adult offenders were excluded since there are no child welfare placements in those institutions. Hence, the total available sample was reduced to a study sample of 354 adolescents (252 child welfare and 102 juvenile justice youths; 223 boys and 131 girls) between 10 and 18 years of age at entry to one of 58 institutions (mean age = 14.5, SD = 1.8). More information on participant demographics by committing authority are printed in Table 1 (see “Results” section).

**Table 1:** Group differences in baseline demographic factors, mental health and history of offending

Characteristic	Total (N = 354)	Welfare Youth (N = 252)	Juvenile Justice Youth (N = 102)	Univariate test of difference	
Demographic factors					
Gender [% male (*n*)]	63.0% (223)	53.2% (134)	87.3% (89)	χ2(1) = 36.18	***
Age mean (SD)	16.02 (1.64)	15.8 (1.55)	16.6 (1.76)	t(320) = -3.95	***
Nationality [% Swiss (n)]	83.1% (294)	85.7% (216)	76.5% (78)	χ2(1) = 4.41	*
Born in Switzerland [% yes (n)]	76.0% (269)	77.8% (196)	71.6% (73)	χ2(1) = 1.53	n.s
Language region of placement				χ2(2) = 8.81	**
German [% (n)]	73.2% (259)	72.6% (183)	74.5% (76)		
French [% (n)]	21.5% (76)	19.8% (50)	25.5% (26)		
Italian [% (n)]	5.4% (19)	7.5% (19)	0.0% (0)		
Age at beginning of current placement mean (SD)	14.95 (1.74)	14.62 (1.72)	15.76 (1.51)	t(350) = -5.89	***
Planned duration of current placement mean (SD)	27.32 21.4	28.05 (22.65)	25.51 (17.93)	t(350) = 1.01	n.s
Type of Institution				χ2(3) = 19.32	***
Transitional Placement	11.9% (42)	15.7% (16)	10.3% (26)		
Group home with school or trade program	58.8% (208)	53.6% (135)	71.6% (73)		
Group home without internal educational program	25.7% (91)	31.7% (80)	10.8% (11)		
Other	3.7% (13)	4.4% (11)	2.0% (2)		
Institutionalization History					
Previous residential or foster placement [% yes (n)]	46.3% (161)	45.2% (112)	49.0% (49)	χ2(1) = 0.42	n.s
Age at first placement mean (SD)	13.46 (3.45)	12.99 (3.67)	14.62 (2.51)	t(269.86) = -4.81	***
Number of previous placements mean (SD)	0.97 (1.38)	0.93 (1.30)	1.09 (1.56)	t(346) = -0.10	n.s
Mental health problems					
Alcohol and drug use mean (SD)	2.87 (2.77)	2.54 (2.70)	3.76 (2.80)	t(320) = -3.55	***
Angry-irritable mean (SD)	4.66 (2.68)	4.66 (2.61)	4.67 (2.87)	t(320) = -0.02	n.s
Depressed-anxious mean (SD)	3.04 (2.39)	3.14 (2.50)	2.75 (2.04)	t(186.61) = 1.46	n.s
Suicide ideation mean (SD)	2.08 (1.70)	2.17 (1.76)	1.82 (1.51)	t(172.39) = 2.24	n.s
More than one elevated MAYSI-2 scale [% yes (n)]	80.1% (258)	80.4% (189)	79.3% (69)	χ2(1) = 0.50	n.s
Violence Exposure and Trauma					
Traumatic Experience [% yes (n)]	84.8% (217)	84.5% (158)	85.5% (59)	χ2(1) = 0.40	n.s
Traumatic experiences mean (SD)	2.4 (1.47)	2.38 (1.42)	2.46 (1.61)	t(320) = -0.42	n.s
Direct victimization mean (SD)	2.04 (1.98)	2.11 (2.08)	1.85 (1.66)	t(188.71) = 1.15	n.s
Self-reported previous delinquency					
General delinquency mean (SD)	8.02 (7.07)	7.04 (6.64)	10.65 (7.55)	t(316) = -4.15	***
Violent delinquency mean (SD)	1.53 (1.83)	1.27 (1.62)	2.23 (2.18)	t(121.19) = -3.74	***
Delinquency severity mean (SD)	2.50 (1.37)	2.30 (1.39)	3.07 (1.15)	t(183.04) = -5.03	***
Previous convictions					
Criminal conviction [% yes (n)]	47.5% (168)	36.9% (93)	73.5% (75)	χ2(1) = 40.75	***
Age at first conviction mean (SD)	13.93 (1.78)	13.91 (1.83)	13.95 (1.73)	t(166) = -0.15	n.s
Violent crime [% yes (n)]	17.5% (62)	8.7% (22)	39.2% (40)	χ2(1) = 46.71	***
Non-violent crime [% yes (n)]	44.4% (157)	34.5% (87)	68.6% (70)	χ2(1) = 34.22	***

All p-values from analyses with continuous data are adjusted for multiple comparisons using a Holm-Bonferroni sequential correction. + Mean age among N = 168 with previous conviction

*** *p* < .001, ** *p* < .01, * *p* < .05

### Measures

#### Predictors

Committing authority was a binary variable specifying whether youth were in a commitment at wave 1 based on child protection reasons by a child welfare authority (civil law measure, N = 252) or by a juvenile criminal court (juvenile justice measure, N = 102).

#### Outcomes

Adult criminal conviction data was obtained from the BFS until the end of 2017, up to 10 years after the initial assessment of the study. We assessed convictions for the two more serious types of offenses (*Verbrechen, Vergehen*), excluding the most minor category of offenses (*Übertretungen*), which under Swiss law are all offenses only punishable by fine (see Art. 103 Swiss Criminal Code). Violent offenses were classified following the definitions used by the BFS and included all offenses that included actual or threatened harm against persons, such as all forms of assault, robbery or coercion. Non-violent offenses were all other offenses above the aforementioned severity threshold, including violence against property or serious drug offenses.

#### Other risk factors for adult criminal convictions

*Gender* was a binary variable coded as 0 = female, 1 = male; *Age at Beginning of Current Placement* was assessed in years.

*Mental health problems and trauma* were assessed using the Massachusetts Youth Screening Instrument-Version 2 (MAYSI-2), one of the most widely used tools for mental health screening for youth entering the juvenile justice system [48]. It consists of a 52-items self-report questionnaire screening for potential emotional or behavioral problems (e.g., suicidal ideation and aggressive behavior) that could require further (psychiatric) evaluation and has shown to be reliable and valid in diverse samples of detained youths [49]. The MAYSI-2 is currently used in detention, intake probation, and/or corrections facilities in about 44 states in the USA as well as a in growing number of institutions in Europe, including Switzerland [48, 50]. In the current study, computerized versions of the French, German and Italian questionnaire were used. Respondents rated all items with yes (1 point) or no (0 points), resulting in seven scales, of which the current study used five [48]: alcohol/drug use, angry-irritable, depressed-anxious, suicide ideation, and traumatic experiences. Thought disturbance and somatic complaints were not included in the current study. Trauma exposure questions vary by gender and are not included in the scoring of cautions and warnings related to the screening.

Data on *previous juvenile convictions* of crimes committed before wave 1 was obtained from the BFS. The information was recoded into a binary variable (yes/no) and again excluded the most minor category of offenses. *Severity of past delinquency* was assessed through a German self-report questionnaire on offending behavior [51]. All analyses were adjusted for *Time at Risk*, which was calculated as time in months that the respondent had been over 18 years of age and thus subject to adult criminal law.

### Statistical analysis

Analyses were performed using Mplus version 8.2 [52] and SPSS version 25 [53]. First, the sample was grouped into two groups of adolescents as described above. Then, univariate difference tests between the two groups were tested either with χ2 difference scores (for categorical variables) or one-way analysis of variance (ANOVA; for continuous variables, see Table 1). All analyses with continuous variables used a Holm-Bonferroni sequential correction to adjust for multiple comparisons and Levene’s correction for unequal variances if necessary [54].

Next, to estimate risk of adult criminal conviction by committing authority we conducted three sets of logistic regressions in Mplus, investigating any, violent and non-violent convictions separately. In each set of regressions, unadjusted models were estimated first, before risk factors (i.e., gender, age at commitment, number of previous convictions, severity of previous self-reported offending, time at risk and time since intake), trauma and mental health treatment needs (alcohol/drug use, angry-irritable, depressed-anxious, suicide ideation) were added to predict outcomes. Multilevel analyses showed intraclass correlations of study variables ranging from 0.08 to 0.57 by placement facility. To account for clustering of the data within facilities, all logistic regression analyses thus used a complex sampling procedure with cluster robust standard errors [55]. Participants with missing data were included in the model estimations using Full Information Maximum Likelihood (FIML) techniques and MLR estimation with Montecarlo integration for binary outcomes was used [56]. Model fit was assessed with the Sample size adjusted Bayesian Information Criterion (BIC) and the Akaike information criterion (AIC).

## Results

### Demographic characteristics of adolescents in residential placements

The first aim of the current study was to examine similarities and differences in demographic markers, current treatment needs (psychiatric profile, substance use) and previous offending (self-report and official conviction) between adolescents placed in residential care by either child protection or juvenile justice authority. As shown in Table 1, results showed that juvenile justice youth were predominantly male, while there was an equal distribution of gender within the welfare youth. There were some geographical differences in placement authority, with an increased proportion of juvenile justice youth coming from the French speaking region of Switzerland compared to welfare youth, while there were no juvenile justice youth from the Italian speaking region. Juvenile justice youth were also older at the time of the study as well as when they were first placed in out-of-home care compared to welfare youth. There were no other differences in institutionalization history or in planned duration of current placement; differences in types of institution are likely due to differences in age and gender between the two groups. There were few differences in current mental health treatment needs and previous self-reported trauma exposure between juvenile justice and welfare youth. However, juvenile justice youth had higher mean levels of self-reported alcohol and drug use. Finally, there were differences in history of offending on both the self-reports as well as official records. Specifically, juvenile justice youth scored higher on all forms of delinquency and had more previous convictions, both violent and non-violent. There were no differences in age of first conviction and importantly, among those who were placed by a juvenile justice authority, 22.5% had no previous criminal conviction.

### Risk of adult criminal conviction by committing authority

To investigate whether the adolescents committed by child welfare or juvenile justice authority differed in their risk for adult criminal conviction we calculated three sets of logistic regressions (second aim). Results of these analyses are presented in Table 2. Unadjusted models showed that committing authority predicted adult criminal conviction overall (model 1a), as well as violent (model 2a) and non-violent convictions (model 3a) separately, with adolescents being committed through juvenile justice authority showing increased risk on all outcomes.

**Table 2:** Logistic regressions predicting adult criminal conviction

Parameter Estimates	OR	(95% CI)		*β*	*S.E*	Est./S.E	
Any Adult Criminal Conviction							
Model 1a							
Committing Authority (0 = child welfare; 1 = juvenile justice)	2.66	(1.47–4.80)		0.24	0.08	2.87	**
AIC, BIC, R^2^	16,876.74			16,893.47		0.06	(n.s.)
Model 1b							
Committing Authority (0 = child welfare; 1 = juvenile justice)	0.93	(0.50–1.71)		− 0.02	0.07	− 0.21	(n.s.)
Gender (0 = female; 1 = male)	6.34	(3.62–11.10)		0.38	0.06	6.27	***
Age at Beginning of Commitment	0.89	(0.68–1.15)		− 0.09	0.12	− 0.79	
MAYSI Traumatic Experiences	1.23	(1.00–1.52)		0.13	0.08	1.57	
MAYSI Alcohol/Drug Use	1.13	(1.03–1.24)		0.15	0.07	2.15	*
MAYSI Angry-Irritable	1.04	(0.91–1.19)		0.04	0.09	0.46	
MAYSI Depressed-Anxious	1.03	(0.84–1.27)		0.03	0.13	0.25	
MAYSI Suicidal Ideation	0.98	(0.82–1.16)		− 0.02	0.08	− 0.25	
Severity of Previous Delinquency	0.89	(0.73–1.10)		− 0.07	0.07	− 0.93	
Previous Conviction	1.90	(1.23–2.96)		0.14	0.06	2.41	*
Time at Risk	1.03	(1.01–1.05)		0.32	0.10	3.27	**
Time since Intake	0.98	(0.96–1.00)		− 0.12	0.08	− 1.45	
AIC, BIC, R^2^	11,865.95			11,905.67		0.40	***

Adult Violent Conviction							
Model 2a							
Committing Authority (0 = child welfare; 1 = juvenile justice)	2.83	1.45	5.51	0.25	0.09	2.73	**
AIC, BIC	16,688.45			16,705.17		0.06	(n.s.)
Model 2b							
Committing Authority (0 = child welfare; 1 = juvenile justice)	0.94	0.51	1.73	− 0.01	0.07	− 0.17	
Gender (0 = female; 1 = male)	4.60	2.04	10.39	0.29	0.10	3.04	**
Age at Beginning of Commitment	0.69	0.47	1.02	− 0.25	0.14	− 1.80	
MAYSI Traumatic Experiences	0.96	0.72	1.29	− 0.02	0.10	− 0.23	
MAYSI Alcohol/Drug Use	1.13	1.00	1.27	0.13	0.08	1.67	
MAYSI Angry-Irritable	0.96	0.81	1.13	− 0.05	0.11	− 0.44	
MAYSI Depressed-Anxious	1.14	0.92	1.41	0.12	0.12	1.01	
MAYSI Suicidal Ideation	0.95	0.76	1.19	− 0.04	0.10	− 0.37	
Severity of Previous Delinquency	0.96	0.71	1.31	− 0.02	0.10	− 0.20	
Previous Conviction	4.32	1.91	9.78	0.29	0.09	3.29	**
Time at Risk	1.04	1.01	1.06	0.36	0.11	3.34	**
Time since Intake	0.96	0.93	0.99	− 0.21	0.09	− 2.38	*
AIC, BIC, R^2^	11,719.86			11,759.58		0.48	***

Adult Non-Violent Conviction							
Model 3a							
Committing Authority (0 = child welfare; 1 = juvenile justice)	2.44	1.27	4.68	0.22	0.09	2.35	*
AIC, BIC, R2	16,858.54			16,875.27		0.05	(n.s.)
Model 3b							
Committing Authority (0 = child welfare; 1 = juvenile justice)	0.86	0.43	1.74	− 0.03	0.08	− 0.34	
Gender (0 = female; 1 = male)	7.10	3.67	13.73	0.40	0.07	5.64	***
Age at Beginning of Commitment	0.86	0.65	1.14	− 0.11	0.12	− 0.92	
MAYSI Traumatic Experiences	1.36	1.12	1.66	0.19	0.08	2.35	*
MAYSI Alcohol/Drug Use	1.09	0.97	1.23	0.10	0.08	1.23	
MAYSI Angry-Irritable	1.06	0.92	1.22	0.07	0.09	0.70	
MAYSI Depressed-Anxious	1.00	0.81	1.24	0.00	0.13	0.02	
MAYSI Suicidal Ideation	0.97	0.81	1.15	− 0.03	0.08	− 0.34	
Severity of Previous Delinquency	0.80	0.65	0.98	− 0.13	0.07	− 1.92	
Previous Conviction	2.04	1.27	3.29	0.15	0.06	2.52	*
Time at Risk	1.03	1.01	1.05	0.32	0.10	3.26	**
Time since Intake	0.97	0.95	1.00	− 0.15	0.08	− 1.84	
AIC, BIC, R^2^	11,852.74			11,892.46		0.43	***

*N* = 354. *** *p* < .001, ** *p* < .01, * *p* < .05 Parameters are standardized; Analyses used cluster-robust standard errors and FIML-Estimation with Montecarlo integration for dichotomous outcomes

However, when controlling for other risk factors of adult criminal conviction, i.e., gender, age at beginning of current placement, severity of previous delinquency, previous conviction, time at risk and time since intake, as well as mental health treatment needs and traumatic experiences (third aim), committing authority no longer had an effect on risk of adult conviction. Specifically, the adjusted models showed that being male and more time at risk was associated with an increased risk for any adult conviction (model 1b) as well as for violent (model 2b) and non-violent convictions (model 3b). Similarly, having a previous conviction increased odds for all forms of adult convictions, while in contrast there was no association between self-reported severity of past delinquency and any of the outcomes. In terms of mental health treatment needs, more alcohol and drug use increased risk of general adult conviction, and traumatic experiences were associated with an increased likelihood of non-violent adult conviction within this high-risk sample. There was no association between trauma, mental health and risk of adult violent conviction.

## Discussion

Adolescents in residential care are marked by multiple disadvantages before and during placement, as well as consequently in young adulthood. They show elevated rates of trauma, psychopathology and other psychosocial problems, and an elevated risk of involvement in both juvenile delinquency and adult criminal behavior [1–4]. At least one third of youth in child welfare are also known to the juvenile justice system [5, 24].

In Switzerland, adolescents are placed in residential care because of delinquent behavior (juvenile justice measure) or for child protection reasons (civil law measure, e.g., maltreatment, neglect, or parental absence, psychopathology or drug abuse), meaning they might reside in the same institutions based on their educational or treatment needs [7, 8]. The current study capitalized on this opportunity to examine effects of shared placement of juvenile offenders with child welfare youth in Switzerland and investigated long-term adjustment in the form of adult criminal conviction. Similarities and differences in demographic markers, current treatment needs (trauma, psychiatric profile, substance use) and previous offending behavior (self-report and official conviction) between both groups were examined, and it was investigated if these demographic and crime-related risk factors, and mental health treatment needs while in residential care influenced risk for adult criminal conviction. Especially investigating the influence of mental health treatment needs on risk of adult offending is of high practical relevance, since it might present an important avenue for intervention.

Results of the current study showed overall few differences in mental health treatment needs between child welfare and juvenile justice youth, and no association between placement authority and risk of adult criminal conviction after accounting for other risk factors and mental health treatment needs. Univariate analyses of group differences showed that, while juvenile justice youth had higher levels of substance use, there were no differences in past traumatic experiences, angry-irritable or depressed-anxious problems on the MAYSI-2. As expected, both groups differed in previous offending behavior as well as in previous convictions, with juvenile justice youth scoring higher on all indicators. It is important to note however, that even among juvenile justice youth, 22.5% had no previous conviction. This indicates that juvenile justice authorities do mandate placements based on educational or treatment needs independent of substantiated delinquent behavior, as intended by Swiss law. The lack of differences in mental health problems or planned duration of placements between the two groups is also an indicator that placement decisions are based mainly on treatment needs and not as a means to discipline juvenile justice youth.

In terms of demographic factors, we found age, gender and nationality differences, as well as some regional differences. While the increased proportion of males among the juvenile justice youth corresponds to international samples of juvenile offenders [1, 34], the age differences and differences in nationality merit some closer attention. While groups did not differ in their number of placements or age at first conviction, juvenile justice youth were older at their first placement as well as at the time of the study. Furthermore, although the results regarding nationality are difficult to interpret,^1^ juveniles with a non-Swiss nationality were more prevalent in the juvenile justice than in the child welfare sample. This could indicate that older adolescents and adolescents with a non-Swiss nationality represent a subgroup where the consequences of dysfunction at home or treatment needs appear later compared to welfare youth whose needs seems to get noticed by authorities earlier. Whether this is associated with the severity of the situation or represents a different reaction of the adolescents to similar scenarios has to be the focus of future research.

Furthermore, the results of the logistic regressions showed several associations between risk factors and adult criminal conviction, as well as differing associations by type of adult criminal conviction. As in the Swiss national data [13], gender was the strongest predictor of adult criminal conviction, with males showing between 4.6 to 7.1 times increased odds for an adult conviction. Same as in the national sample, in the present high-risk population previous juvenile convictions were associated with an increased likelihood of general, violent and non-violent conviction. However, these findings were not repeated in self-reports of juvenile delinquency, with self-reported severity of previous delinquency having no association with any of the outcomes when other factors were taken into account. While our results showed that placement authority did not influence likelihood of adult conviction after accounting for other risk factors, this difference indicates that there might be labeling processes by contact with the juvenile justice system that results in legal convictions. However, they do not seem to influence reasons for institutionalization. Further investigation of mechanisms behind these results should be the focus of future research.

Lastly, our results showed an association between traumatic experiences and non-violent adult conviction up to ten years later, as well as an association between alcohol and drug use and general adult conviction. The latter is consistent with the results of previous studies in which substance use problems were found to be related to (adult) (re)offending [57–60]. Despite a small effect size, it could still be important to screen for alcohol and drug problems in adolescents upon entering the residential care institution, so these problems can be taken into account in treatment in order to prevent long-term negative outcomes, such as delinquency in adulthood [59, 61, 62]. Regarding trauma, though the effect sizes were also comparatively small, our findings correspond to the body of research showing a connection between childhood trauma, delinquency and adult criminal involvement [63–65], whilst noting that this does not apply to all forms of childhood traumatic experiences [66]. Hence traumatic experiences and psychosocial stress should also be included into standard screening and assessment, and taken into account in the treatment of juvenile and adult offenders [66]. Evidence-based trauma-therapeutic interventions as well as trauma-pedagogic care concepts should be embedded into child welfare and juvenile justice settings. Trauma-informed care is a conceptual framework and milieu therapeutic approach that relates to the understanding of and responsiveness to trauma exposure [67]. It conceptualizes problem behaviour in the context of an individual’s traumatic exposure and contains anticipating and avoiding practices which increase the risk of traumatic re-enactment [68, 69]. Guiding principles of trauma informed care include: safety, trustworthiness and transparency, peer support, collaboration and mutuality, empowerment and choice, and cultural, historical and gender issues (see also the infographic on the website of the Office of Public Health Preparedness and Response [OPHPR] of the Center for Disease and Control Prevention [CDC]: https://www.cdc.gov/cpr/infographics/6_principles_trauma_info.htm).^2^ This trauma informed approach has also been examined in the more specific context of the juvenile justice system [70, 71]. Although the combination of evidence-based trauma-therapeutic interventions in combination with trauma informed care concepts is highly promising for the treatment of, for example juvenile offenders (but also adolescents in the child welfare system), more research is warranted to examine its impact on offending behavior/recidivism as well as other adolescent/adult functional outcomes.

### Limitations

The current study must be seen in the light of several limitations. A first set of limitations relate to the research design of the larger MAZ. study from which this sample was drawn [8]. First, the classification child welfare versus juvenile justice youth was based on the placement ground in the institution at baseline assessment of the study. However, research in the field of crossover youth has shown that a percentage of adolescents appear in both systems during their childhood/young adulthood [5]. Second, in our sample, we know that a part of the adolescents was in out-of-home care before and could still be found in both systems after the study. Unfortunately, we were unable to carry out a comprehensive, accurate residential care trajectory analysis, on the one hand because the adolescents are not always fully aware of their history and on the other hand because this information is not collected in a structured manner by a centralized organization in Switzerland. Finally, by design, participants were interviewed at varying time points after the beginning of their institutional stay. The MAYSI-2 however is designed to be administered at intake into a juvenile justice facility. Given the time limited nature of the anchoring questions in this screening measure, it can therefore not be excluded that the results have been influenced by the varying time spent already in an institution. We tried to offset this limitation by controlling for time since intake in our analyses.

A second set of limitations concerns the assessment used in the current study. An important point is that many of the tools we used in this study were self-report instruments (MAYSI-2, self-reported delinquency). The use of self-report instruments entails a risk of both overestimation and underestimation. On the other hand, it offers the opportunity to gain more insight into certain aspects (often relating to internalizing mental health) that may have been overlooked when using only third-party assessments. Notably, we used official registered criminal convictions for the outcome variables. However, future research and analyses should include information from multiple sources. Finally, trauma is a broad and multi-faceted concept with often no clear definition leading to an exponential use. We used the traumatic experience scale of the MAYSI-2, which is a very rudimentary screening scale only consisting of a limited number of items. This approach takes little account of the number, duration or effect of a certain (potentially) traumatic experience and is supposed to be a quick screening tool that needs further enhanced clarification and more sophisticated measurement tolls. Nevertheless, it is a short and feasible indicator for possible trauma exposure.

## Conclusion

Our results support the approach of placement in residential care institutions based on treatment needs in this Swiss sample. Adolescents’ reason for placement were unrelated to risk of adult criminal conviction when taking into account well documented demographic risk factors (male gender, previous conviction and more time at risk). In addition, there was an effect of trauma histories and mental health needs beyond these static factors, indicating a possible avenue for intervention for all adolescents. Our results thus underscore the importance of assessing trauma and mental health status of all adolescents entering residential or other out-of-home placement and addressing their treatment needs, with a special attention to trauma-informed care. Finally, although it is impossible to make general statements given the differences in legal systems, countries might reflect on whether they want to place adolescents strictly based on the adjudicating court or whether they want to take the (underlying) problems of these youngsters into account. This seems especially pertinent for the high percentage of “cross-over youths”, adolescents who have records in both systems.

## Data Availability

The data that support the findings of this study are available from the corresponding author upon request.

## Footnotes

1 The results regarding nationality are difficult to interpret for various reasons. Firstly, (cultural/ethnic) minority groups are defined in the international literature in various ways, e.g., race, ethnicity, nationality, country of birth. Secondly, as far as we know, there are no numbers on nationality among child welfare youths in Switzerland. Numbers from the Swiss Federal Statistical Office show that in 2010 about 21% of young people between the ages of 10 and 18 had a non-Swiss nationality (https://www.pxweb.bfs.admin.ch/pxweb/de/px-x-0102020000_103/px-x-0102020000_103/px-x-0102020000_103.px). As far as convictions and residential placement are concerned, this percentage was 29% (https://www.bfs.admin.ch/bfs/de/home/statistiken/kriminalitaet-strafrecht/strafjustiz/jugend-erwachsenenurteile.assetdetail.8946454.html) and 36% (https://www.bfs.admin.ch/bfs/de/home/statistiken/kriminalitaet-strafrecht/justizvollzug/platzierte-jugendliche.assetdetail.8947222.html) respectively for this age group in the same year. Our results regarding non-Swiss nationality of the juvenile justice sample are lower than the conviction and residential placement rates of the Swiss Federal Statistical Office. This could be due to the fact that some juveniles with a non-Swiss nationality were unable to complete the questionnaires due to insufficient knowledge of the French, German and Italian language and were therefore excluded from participation. The same could apply to the child welfare sample, which, with 14% of non-Swiss nationals, is underrepresented in relation to the general population.

2 It should be taken into account that the concept of trauma-informed care is still under development and is being interpreted in different ways by various authors and agencies.
